# Mindfulness may both moderate and mediate the effect of physical fitness on cardiovascular responses to stress: a speculative hypothesis

**DOI:** 10.3389/fphys.2014.00105

**Published:** 2014-03-25

**Authors:** Marcelo M. P. Demarzo, Jesús Montero-Marin, Phyllis K. Stein, Ausiàs Cebolla, Jaime G. Provinciale, Javier García-Campayo

**Affiliations:** ^1^Department of Preventive Medicine, “Mente Aberta” – Brazilian Center for Mindfulness and Health Promotion, Universidade Federal de São PauloSão Paulo, Brazil; ^2^Department of Psychiatry, Instituto Aragonés de Ciencias de la Salud de la Red de Actividades Preventivas y de Promoción de la Salud (REDIAPP), Universidad de ZaragozaZaragoza, Spain; ^3^Heart Rate Variability Laboratory, Division of Cardiology, Internal Medicine Department, Washington University School of MedicineSaint Louis, MO, USA; ^4^Department of Basic and Clinical Psychology and Psycholobiology, Universitat Jaume ICastellón, Spain; ^5^Centro de Investigación Biomédica en Red - Fisiopatología de la Obesidad y Nutrición (CIBERobn)Madrid, Spain; ^6^Human Centered Technology Department, Instituto en Bioingeniería y Tecnología Orientada al Ser Humano, Universitat Politècnica de ValènciaValencia, Spain

**Keywords:** mindfulness, moderator effects, mediational model, physical fitness, cardiovascular system, psychological stress, mindful exercise

## Abstract

The psychological construct of mindfulness refers to an awareness that emerges by intentionally paying attention to the present experience in a non-judgmental or evaluative way. This particular quality of awareness has been associated to several indicators of physical and psychological health, and can be developed using mindfulness-based interventions (MBIs), and therefore MBIs have been successfully applied as preventive and complementary interventions and therapies in medicine and psychology. Together with quiet sitting and lying meditation practices, mindful physical exercises such as “mindful walking” and “mindful movement” are key elements in MBIs and couple muscular activity with an internally directed focus, improving interoceptive attention to bodily sensations. In addition, MBIs seem to share similar mechanisms with physical fitness (PF) by which they may influence cardiovascular responses to stress. Based on these facts, it is feasible to raise the question of whether physical training itself may induce the development of that particular quality of awareness associated with mindfulness, or if one's dispositional mindfulness (DM) (the tendency to be more mindful in daily life) could moderate the effects of exercise on cardiovascular response to stress. The role of mindfulness as a mediator or moderator of the effect of exercise training on cardiovascular responses to stress has barely been studied. In this study, we have hypothesized pathways (moderation and mediation) by which mindfulness could significantly influence the effects of PF on cardiovascular responses to stress and discussed potential practical ways to test these hypotheses.

## Mindfulness in medicine and psychology

Interest in mindfulness has increased exponentially in recent decades in academic and clinical contexts (Dimidjian and Kleiber, 2013). The psychological construct of mindfulness refers to an awareness that emerges by intentionally paying attention to the present experience in a non-judgmental way (Kabat-Zinn, 2003). In other words, a “mindful” mind brings together attentional and attitudinal features at the same time, self-regulating attention toward present-moment direct experiences and attitude in a non-judgmental tone toward internal and external phenomena (physical, affective and behavioral).

This particular quality of non-evaluative awareness can improve one's physical and psychological health status, and therefore several approaches generally called “mindfulness-based interventions” (MBIs) have been developed and tested in the last 40 years (Cullen, 2011). Evidence about their efficacy and effectiveness in improving mindfulness and health is rapidly accumulating (Khoury et al., 2013).

MBIs, e.g., “Mindfulness-based Stress Reduction” (MBSR) and “Mindfulness-based Cognitive Therapy” (MBCT), are generally 8-weeks group-based interventions that mix mindfulness practices (mostly derived from ancient eastern meditative techniques) with contemporary cognitive and behavioral approaches to improve one's mindfulness levels and capabilities to adaptively manage stressful life events (Cullen, 2011). Moreover, MBIs have been successfully applied as preventive and adjuvant therapies in patients with depression, anxiety, chronic pain, cardiovascular disorders, cancer, and other non-communicable diseases (Bonadonna, 2003; Edelman et al., 2006; Kuyken et al., 2008; Fortney and Taylor, 2010; Khoury et al., 2013). In addition, MBIs have been adapted to special targeted population (Carson et al., 2006; Duncan and Bardacke, 2010; Cullen, 2011), including the sports psychology field, in this case aiming to improve athletes' psychological well-being and to enhance their performance (Gardner and Moore, 2012).

It has been suggested that MBIs result in improvements in autonomic and central nervous systems regulation, attention control, emotional and behavioral self-awareness and regulation, self-compassion, resilience, dispositional flow, and body awareness, and these mechanisms may account for their efficacy (for reviews about mindfulness mechanisms see Hölzel et al., 2011; Gardner and Moore, 2012).

## Mindfulness: trait and state

Several questionnaires and scales to measure mindfulness trait or state have been developed in the last two decades (Park et al., 2013), which allow systematic investigation in this field. It has been observed that to some degree the particular type of awareness associated to mindfulness may be innate (Garland et al., 2013).

Trait or dispositional mindfulness (DM), the tendency to be more mindful in daily life (Garland et al., 2013), seems to be a sum of individual genetics and life experiences, and DM is not necessarily related to having participated in an MBI or practicing meditative exercises. DM can be measured by scales addressing mindfulness trait, e.g., the Mindful Attention Awareness Scale (MAAS) (Brown and Ryan, 2003) and the Five Facets Mindfulness Questionnaire (FFMQ) (Bohlmeijer et al., 2011), and it has been significantly related to several indicators of psychological and physical health including: higher levels of positive affect, improvement in personal stress management skills, and in adaptive emotional regulation (Hayes-Skelton and Graham, 2013; Khoury et al., 2013; Garcia-Campayo et al., 2014).

As expected, DM can be also modulated by participating in MBIs and practicing specific mindfulness techniques such as several types of meditative practices (Garland et al., 2010; Park et al., 2013). These interventions can induce transient increases in the mindfulness state, and may promote long-term improvements in DM if applied in a regular basis (Soler et al., 2014). The “mindfulness state,” i.e., mindfulness addressed as a statelike mental behavior, which is context-dependent, transient and variable, is also measureable, and few scales have been developed for this purpose (Tanay and Bernstein, 2013).

## Mindfulness and cardiovascular responses to stress

Chronic stress is a well-known risk factor for cardiovascular diseases (CVD). Physical and psychological stressors can provoke non-adaptive stress-induced cardiovascular responses marked by dysfunctional cortisol and catecholamine releasing, systemic inflammation, oxidative stress, and unbalanced autonomic nervous system, leading to endothelial dysfunction, increased blood pressure, among others factors, which chronically may induce hypertension and CVD (Koolhaas et al., 2011; Huang et al., 2013; Stoner et al., 2013). Current evidence suggests that MBIs may buffer responses to stressors by mechanisms direct or indirectly related to these biomarkers of non-adaptive cardiovascular responses to stress.

A recent study showed that an MBSR program (the original program that have influenced subsequent types of MBIs) had a favorable influence both on biomarkers of stress regulation, such as cortisol secretion and sleep (Brand et al., 2012). In this regard, stress-sleep connection may be an important mechanism influencing responses to stress (Demarzo and Stein, 2012). In the same direction, another study showed that an MBI resulted in increases in both objectively- and subjectively-measured sleep continuity in patients using anti-depressant medication (Britton et al., 2012).

MBIs and other behavioral interventions designed to reduce emotional reactivity may be of therapeutic benefit in chronic inflammatory conditions (Rosenkranz et al., 2013). Even a brief mindfulness intervention in the workplace may be an effective and probably cost-effective way to reduce systemic inflammation (Malarkey et al., 2013). MBSR has also shown promise as a novel treatment approach for reducing loneliness-related pro-inflammatory gene expression in older adults (Creswell et al., 2012). Moreover, analysis of oxidative stress levels in people who meditate indicated that meditation correlates with lower oxidative stress and higher melatonin levels, a well-known antioxidant agent (Martarelli et al., 2011).

Another mechanism that may link mindfulness to cardiovascular responses to stress is influencing on the autonomic nervous system, the body's most primitive and automatic regulator of the stress responses (Thayer et al., 2010). One way to measure the functioning of the autonomic nervous system is to analyze heart rate variability (HRV). It is well-known that depressed HRV, suggesting a lack of flexibility of autonomic control, has been linked to stress factors (Thayer et al., 2010). Recent studies have shown the potential of mindfulness practices to improve HRV, e.g., increasing the parasympathetic tone (Peressutti et al., 2011; Libby et al., 2012; Krygier et al., 2013).

Furthermore, it was observed in a recent study that MBSR helped reduce blood pressure levels and blood pressure reactivity to stress among healthy community-dwelling individuals reporting elevated stress levels (Nyklíček et al., 2013a). In addition, Prakhinkit et al. (2013) observed in a randomized controlled study that both walking meditation (a key mindfulness practice in MBIs) and traditional walking exercise may improve endothelium-dependent vasodilation in elderly with depressive symptoms (Prakhinkit et al., 2013).

Moreover, MBIs may improve both the cardiovascular response to stressors and provide a complementary approach for the prevention and treatment of CVD. A large study showed that a multidimensional intervention based on mindfulness and other integrative medicine principles reduced the 10-year risk of coronary heart disease (CHD) for outpatients with 1 or more known cardiovascular risk factors when compared to usual care, although the effect of the specific components of the intervention were not tested separately (Edelman et al., 2006). Furthermore, a MBSR program may reduce characteristics of the distressed personality (‘Type D), and this effect seems to be mediated by increase in self-reported mindfulness (Nyklíček et al., 2013b). The “Type D” personality is characterized by a combination of negative affectivity and social inhibition, and has been associated with adverse health outcomes, including CVD (Nyklíček et al., 2013b).

## Hypothesis rationale

Together with sitting and lying meditation practices, mindfulness exercises such as “mindful walking” and “mindful movements” are key elements in MBIs and couple muscular activity with an internally directed focus (Kabat-Zinn, 2005), improving interoceptive attention to bodily sensations (Ospina et al., 2007; Posadzki and Jacques, 2009; Farb et al., 2013; Gryffin and Chen, 2013; Hagins et al., 2013). This may suggest that a regular physical activity made with internal focus may also improve one's mindfulness levels (Tsang et al., 2008).

**Figure 1:**
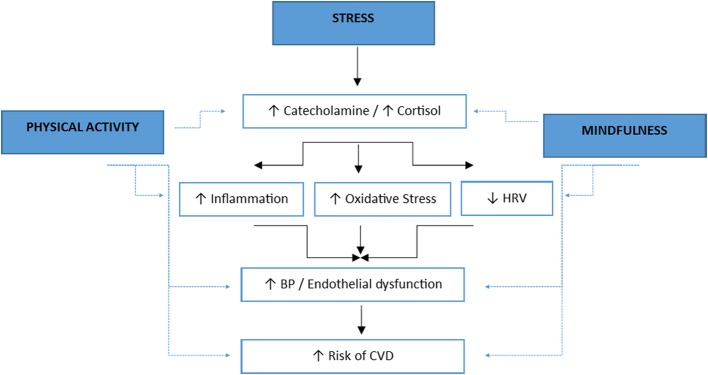
**Potential physiological mechanisms shared by physical activity and mindfulness by which they may attenuate cardiovascular responses to stress (the solid line indicates an activation of the indicated factor and the dashed line represents the attenuation of the indicated factors)**. Adapted from Huang et al. (2013). HRV: heart rate variability. BP: blood pressure. CVD: cardiovascular disease.

In addition, as shown in the previous section, MBIs may buffer cardiovascular responses to stress probably sharing similar mechanisms by which physical fitness (PF) influence these responses (Huang et al., 2013; Stults-Kolehmainen, 2013). Figure 1 schematically represents potential physiological mechanisms shared by physical activity and mindfulness by which they may attenuate cardiovascular responses to stress (Huang et al., 2013).

Based on these facts, it is feasible to raise the question of whether physical training itself may induce the development of that particular quality of awareness associated with mindfulness (mindfulness as a mediator variable), or if one's DM could moderate the effects of exercise on cardiovascular response to stress. Until now, there has been a lack of information in scientific literature regarding the role of mindfulness as a mediator or moderator of effects of exercise training on stress and health in general, and on cardiovascular responses to stress in particular. Thus, in this study we aimed to develop the hypothesis that mindfulness influences the effects of PF on cardiovascular responses to stress by both moderating or mediating these effects, and discussed potential practical ways to test these hypotheses.

## Mindfulness as a moderator of the effects of exercise on cardiovascular response to stress

**Figure 2:**
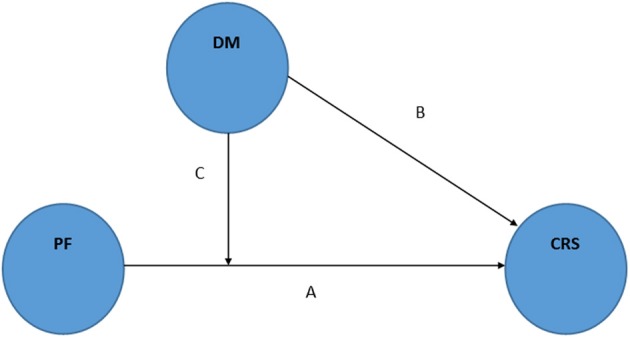
**Schematic representation of the moderator hypothesis: mindfulness as moderator of the physical fitness effects on cardiovascular responses to stress**. Adapted from Baron and Kenny (1986). PF: physical fitness. DM: dispositional mindfulness. CRS: cardiovascular responses to stress.

As represented in the Figure 2 (line “C”), our first hypothesis is that DM acts as a moderator in the association between PF (independent variable) and cardiovascular responses to stress (dependent variable), by altering the strength of the association. By considering both PF and DM as continuous variables (Baron and Kenny, 1986), probably the moderation occurs in a linear direction (the more DM, the more improvement on dysfunctional stress-induced cardiovascular reactivity caused by PF). Thus, we may theorize that exercise training affects differently people with higher or lower DM.

Although there is no published data about this potential moderator role of DM, it is feasible to speculate on it as mindfulness may be seen as an “independent variable” (see Figure 2, line “B”) in the relationship with the “dependent variable” cardiovascular response to stress, an important pre-requisite to be considered as a moderator factor (Baron and Kenny, 1986). In addition, it has been suggested that sex, obesity levels, and individual's perception of control over a situation may be moderators of the effects of PF (Huang et al., 2013), and so DM could be another factor to be taken in account in future studies. Moreover, it is interesting to note that some authors have discussed the idea that only less stressed people could benefit from exercise effects (Stults-Kolehmainen, 2013), and so DM could be a potential confounder factor in this phenomena, as it is well-known that DM is related to less perceived stress and stress-induced biomarkers (Ciesla et al., 2012; Murphy et al., 2012).

In addition, recent findings have identified variables potentially associated with DM that may be confounding variables for the effects of PF on cardiovascular responses to stress. Lower or higher DM may mean lower or higher levels of psychological well-being, healthy behaviors, adherence to an exercise program, emotional reactivity, social anxiety, and co-morbidity, and so may alter the strength of the association between exercise training and cardiovascular responses to stress (Ulmer et al., 2010; Bränström et al., 2011; Salmoirago-Blotcher et al., 2011; Ciesla et al., 2012; Brown et al., 2013; Garcia et al., 2014). It matters mainly if investigators are not using a randomized controlled design to study that association. Even in this case, stratified randomization for DM may be of interest in order to prevent this potential bias.

**Figure 3:**
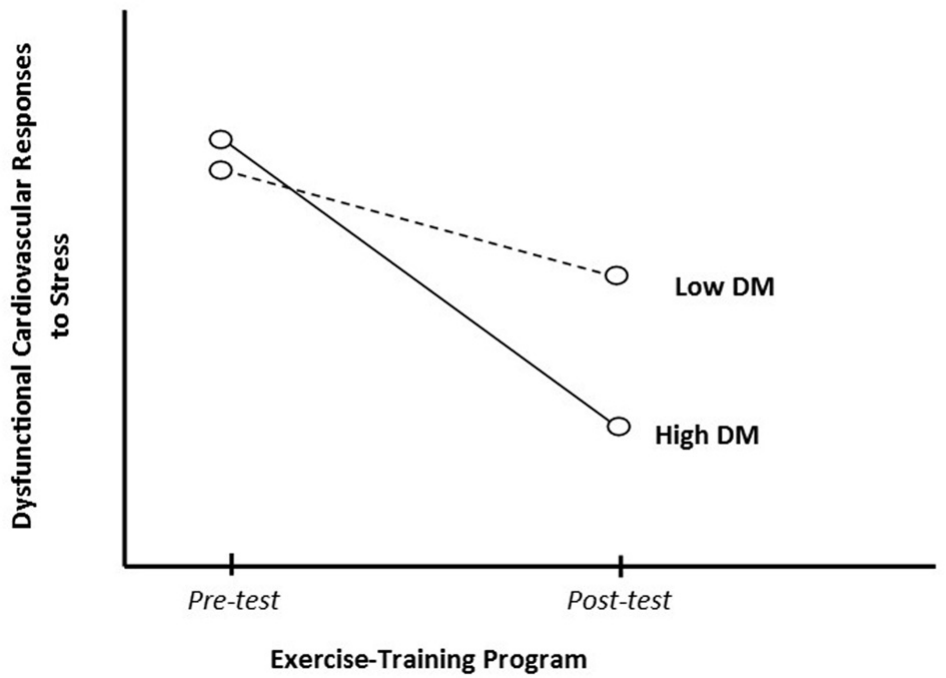
**Dispositional mindfulness (DM) as moderator of the physical fitness effects on cardiovascular responses to stress**.

In order to test the hypothesis that DM is a moderator of the effects of PF on cardiovascular responses to stress, an investigator should address this fundamental research question: does an exercise-training program produce more benefits for people with higher levels of DM but fewer for those with lower levels? If it was the case, it would be expected to find an interaction as hypothesized in Figure 3, where is shown potential additive or synergistic effects between PF and DM. To test such a question, the investigator should pre-specify DM as a moderator variable of interest before the intervention is delivered, and should observe whether DM influences the strength of the association between the exercise training intervention and outcomes related to cardiovascular responses to stress. In other words, to test whether the causal relation between PF and cardiovascular responses to stress changes as a function of DM (Baron and Kenny, 1986). In statistical terms, the slope of the interaction between DM and the exercise-training program improving PF, in a multivariate regression model, should be significant.

## Mindfulness as a mediator of the effects of exercise on cardiovascular response to stress

**Figure 4:**
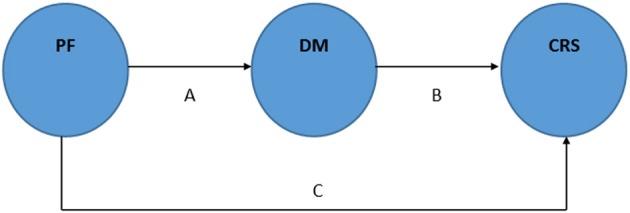
**Schematic representation of the mediator hypothesis: mindfulness as mediator (A + B) of the physical fitness effects on cardiovascular responses to stress**. Adapted from Baron and Kenny (1986). PF: physical fitness. DM: dispositional mindfulness. CRS: cardiovascular responses to stress.

Our second hypothesis is that mindfulness may be useful to understand the processes through which PF influences changes in cardiovascular responses to stress (i.e., mindfulness as a mediator mechanism of PF effects). In this case, rather than hypothesizing a direct causal relationship between the exercise training and outcomes, our mediational model hypothesizes that exercise training causes changes on mindfulness levels, which in turn helps to account for outcomes related to cardiovascular responses to stress (Figure 4, lines “B” plus “C”).

Two recent studies give support to this mediational hypothesis. Goldin and colleagues (Goldin et al., 2012) using a randomized controlled design observed, among other findings, that both MBI and an 8-week aerobic exercise (AE) program were with reductions in self-reported negative emotional reactivity to negative self-beliefs. Although it was not their primary outcome, interestingly, they also observed that AE increased participants' mindfulness levels measured by the Kentucky Inventory of Mindfulness Skills (Baum et al., 2010). This increase was statistically significant but less intense than that induced by the MBI, although there were no significant differences between groups in pre-to-post change (Goldin et al., 2012). Another study, led by Zgierska et al. (2013), interestingly showed that increases in DM measured by MAAS seems to be a better predictor than exercise intensity in preventing cold illness severity when comparing a MBI to an AE program to prevent acute respiratory infection, using a randomized controlled design (Zgierska et al., 2013). Together, these findings suggest that a “classical” AE program also has the potential to promote mindful awareness, and the hypothesis that mindfulness is a mediator of the exercise training on cardiovascular responses to stress may be feasible.

A potential mechanism that could explain this finding is that during and just after (recovery period) a physical exercise, mainly of moderate to vigorous intensity, it is common the awareness of our present moment body sensations such as our breathing rhythm, body temperature, or some kind of body transient momentary discomfort, or even some changing in our internal affective state. Such a combination may enhance one's acute mindfulness state, and chronically one's DM. This has resemblance with the cross-stressor adaptation hypothesis that postulates that there is not only a direct adaptation to the exercise load but also a transfer to everyday life stress situations (Sothmann et al., 1996).

To confirm the mediational hypothesis presented herein, an investigator should test whether an exercise program produces the observed outcomes in cardiovascular responses to stress partially via changes in mindfulness levels (mediator variable). In general, to test mediation hypotheses, it is common to use statistical methods such as regression analysis in order to verify whether both the intervention (exercise program) and the outcome (cardiovascular responses to stress) covary as expected with the hypothesized mediating variable (mindfulness levels), and whether controlling for mindfulness levels explains part of the exercise program effects (Baron and Kenny, 1986). In other words, it is necessary to demonstrate that the relationship between the exercise program and the cardiovascular responses to stress is significantly lower when the mindfulness level is included in the equation. Using the representation in Figure 4, when paths “A” and “B” are controlled, a previously significant relation between the independent (PF) and dependent variable (cardiovascular responses to stress) is less or no longer significant (Baron and Kenny, 1986).

## Mindfulness as both a moderator and mediator of the effects of exercise on cardiovascular response to stress

**Figure 5:**
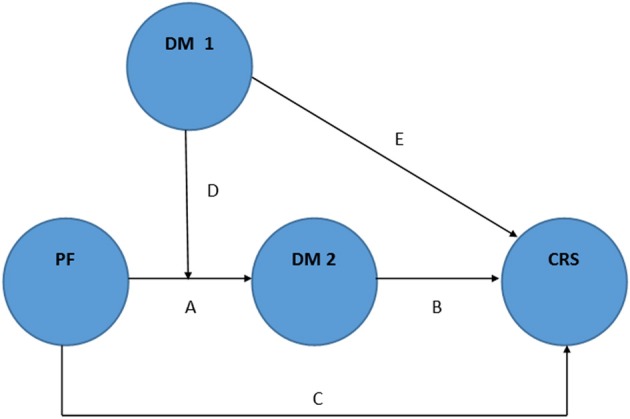
**Schematic representation of the general hypothesis: mindfulness as both moderator and mediator of the physical fitness effects on cardiovascular responses to stress (“D” represents “mediated moderation”)**. Adapted from Baron and Kenny (1986). PF: physical fitness. DM: dispositional mindfulness. CRS: cardiovascular responses to stress. DM 1: DM pre-intervention. DM 2: DM post-intervention.

Our third hypothesis is a combined model with mindfulness having both mediator and moderator status (Figure 5). Baron and Kenny coined the term “mediated moderation” for this potential kind of relation among variables (Baron and Kenny, 1986). In Figure 5, mediated moderation would be indicated by “PF × DM1” affecting “CRS” in line “D,” and “PF × DM1” affecting “DM2” and “DM2” affecting “CRS” in lines “D” plus “B”. So it is possible for “DM2” (post-intervention DM) to mediate both the effect of PF on cardiovascular responses to stress, and the effect of “PF × DM1” (pre-intervention DM moderating PF) on that outcome.

It may be explained by the fact that pre-intervention DM levels (DM1) may differentially affect post-intervention DM (DM2) induced by PF (“mediated moderation”). In other words, one's pre-intervention DM moderates the effects of PF on post-intervention DM (DM2) levels, and then DM2 mediates the effects of PF on cardiovascular responses to stress (dependent variable). Once more, there is no direct evidence about this hypothesis, but it has been suggested that individuals with higher pre-intervention DM have greater benefits (e.g., higher post-intervention DM) from participating in a MBI (Shapiro et al., 2011), and the same process may occur regarding the effects of an exercise intervention. To test this hypothetical model, researchers might use modeling of covariance structures.

## Implications for future studies

Based on our hypotheses, some further studies could be performed. One possibility would be to compare results from AE programs done with additional mindfulness training to usual training (non-mindful component) using the mediational hypothesis analysis. Another study, maybe the most innovative type, would be to compare an exercise session performed with additional instruction to enhance mindfulness, i.e., with full attention to movements and bodily sensations, to a usual training session without this kind of instruction. This last possibility was also suggested by Stults-Kolehmainen (2013, p. 3). In this same study, another possibility for control group would be individuals that intentionally do not stay aware of the exercise, as it has been suggested that multitasking during exercise results in less beneficial stress adaptations (Breus and O'Connor, 1998).

With regard to this last potential study, there are few well-designed studies that have already compared mindful modes of exercise to non-mindful ones but not specifically related to cardiovascular responses to stress (Netz and Lidor, 2003; Tsang et al., 2008). Although an initial study had ambiguous results (Brown et al., 1995), one interesting study examined the effect of a single session of mindful exercise on mood. The intervention compared four physical exercise modes: yoga, the Feldenkrais technique (awareness through movement), aerobic dance, and swimming, with a cognitive exercise delivered by computer lessons serving as control. Measures of mood improved following Feldenkrais, swimming, and yoga, but did not improve following aerobic dance and computer lessons (Netz and Lidor, 2003), supporting the idea that mindful exercise may have additional benefits compared to non-mindful modes of exercises.

In addition, future studies should examine some competing theories as alternatives to our hypotheses. One possibility is that higher scores in DM could help persons to better perform or tolerate exercise training (e.g., through better training psychological and physiological adaptation), consequently improving adherence and compliance to exercise protocols. Another competing argument would be that exercise training might not result in an increase in DM, but that exercise training could improve certain factors such as perceived stress (Starkweather, 2007) which are also improved by the practice of mindfulness, and so both would have positive effects, but exercise would not necessarily lead to improvements in DM.

As expected, our theoretical model has limitations. A main limitation is that scales and questionnaires to measure mindfulness are still object of scientific debate, and some authors have argued that they lack construct validity in measuring the complexity of mindfulness (Grossman et al., 2011; Park et al., 2013). In this way, future situational measurements of mindfulness (Mitchell et al., 2013) could be more effective to support or reject our hypotheses. Another main limitation is that mindfulness seems to be a multidimensional construct (Grossman et al., 2011; Park et al., 2013) and we have not addressed this fact herein in order to simplify the model. In this direction, further research should address the differential moderator and mediator roles of these mindfulness sub-dimensions, e.g., those addressed by the FFMQ (“observing,” “describing,” “acting with awareness,” “non-judging of inner experience,” and “non-reactivity to inner experience”) (Bohlmeijer et al., 2011).

## Conclusion

In conclusion, we have developed the hypothesis that mindfulness may both moderate and mediate the effects of PF on cardiovascular response to stress. This innovative structural model may oxygenate the debate in this research field and help researchers better understand the effects of PF on cardiovascular responses to stress. In addition, general research on both fields of exercise and mindfulness may benefit from this kind of theoretical approach as well.

Conceivably, a “mindful” exercise may provide benefits that are not available in “non-mindful” regular exercise or in opposition to situations where exercise is performed in a multitasking context (Breus and O'Connor, 1998). If so, a mixture of aerobic or resistance physical training with mindfulness would have the potential to improve cardiovascular response to stress in a more effective direction.

Based on these feasible hypotheses, exercise researchers should consider mindfulness in their future research protocols in order to address questions such as whether a higher DM is associated with better health outcomes in an exercise protocol, or if a mindfulness training session mixed into an exercise program could improve health and physical performance among patients, general population, and athletes.

## Author contributions

Marcelo M. P. Demarzo presented the initial concept of the hypothesis, drafted the first version, and organized subsequent versions until the final format of the manuscript. Jesús Montero-Marin, Phyllis K. Stein, Ausiàs Cebolla, Jaime G. Provinciale, and Javier García-Campayo contributed to the development and improvement of the hypothesis and manuscript until their final version.

### Conflict of interest statement

The authors declare that the research was conducted in the absence of any commercial or financial relationships that could be construed as a potential conflict of interest.

## References

[B1] Baron R. M. Kenny D. A. (1986). The moderator–mediator variable distinction in social psychological research: conceptual, strategic, and statistical considerations. J. Pers. Soc. Psychol. 51, 1173–1182 10.1037/0022-3514.51.6.1173 3806354

[B2] Baum C. Kuyken W. Bohus M. Heidenreich T. Michalak J. Steil R. (2010). The psychometric properties of the Kentucky Inventory of Mindfulness Skills in clinical populations. Assessment 17, 220–229 10.1177/1073191109356525 20040728

[B3] Bohlmeijer E. ten Klooster P. M. Fledderus M. Veehof M. Baer R. (2011). Psychometric properties of the five facet mindfulness questionnaire in depressed adults and development of a short form. Assessment 18, 308–320 10.1177/1073191111408231 21586480

[B4] Bonadonna R. (2003). Meditation's impact on chronic illness. Holist. Nurs. Pract. 17, 309–319 14650573 10.1097/00004650-200311000-00006

[B5] Brand S. Holsboer-Trachsler E. Naranjo J. R. Schmidt S. (2012). Influence of mindfulness practice on cortisol and sleep in long-term and short-term meditators. Neuropsychobiology 65, 109–118 10.1159/000330362 22377965

[B6] Bränström R. Duncan L. G. Moskowitz J. T. (2011). The association between dispositional mindfulness, psychological well-being, and perceived health in a Swedish population-based sample. Br. J. Health Psychol. 16(Pt 2), 300–316 10.1348/135910710X501683 21489058 PMC3762484

[B7] Breus M. J. O'Connor P. J. (1998). Exercise-induced anxiolysis: a test of the “time out” hypothesis in high anxious females. Med. Sci. Sports and Exerc. 30, 1107–1112 10.1097/00005768-199807000-00013 9662680

[B8] Britton W. B. Haynes P. L. Fridel K. W. Bootzin R. R. (2012). Mindfulness-based cognitive therapy improves polysomnographic and subjective sleep profiles in antidepressant users with sleep complaints. Psychother. Psychosom. 81, 296–304 10.1159/000332755 22832540 PMC3495555

[B9] Brown D. R. Wang Y. Ward A. Ebbeling C. B. Fortlage L. Puleo E. (1995). Chronic psychological effects of exercise and exercise plus cognitive strategies. Med. Sci. Sports Exerc. 27, 765–775 10.1249/00005768-199505000-00021 7674883

[B10] Brown K. W. Goodman R. J. Inzlicht M. (2013). Dispositional mindfulness and the attenuation of neural responses to emotional stimuli. Soc. Cogn. Affect. Neurosci. 8, 93–99 10.1093/scan/nss004 22253259 PMC3541486

[B11] Brown K. W. Ryan R. M. (2003). The benefits of being present: mindfulness and its role in psychological well-being. J. Pers. Soc. Psychol. 84, 822–848 10.1037/0022-3514.84.4.822 12703651

[B12] Carson J. W. Carson K. M. Gil K. M. Baucom D. H. (2006). Mindfulness-Based Relationship Enhancement (MBRE) in couples, in Mindfulness-Based Treatment Approaches: Clinician's Guide to Evidence Base and Applications, ed Carson J. W. (Durham, NC: Elsevier Academic Press), 309–331 10.1016/B978-012088519-0/50015-0

[B13] Ciesla J. A. Reilly L. C. Dickson K. S. Emanuel A. S. Updegraff J. A. (2012). Dispositional mindfulness moderates the effects of stress among adolescents: rumination as a mediator. J. Clin. Child Adolesc. Psychol. 41, 760–770 10.1080/15374416.2012.698724 22775559

[B14] Creswell J. D. Irwin M. R. Burklund L. J. Lieberman M. D. Arevalo J. M. G. Ma J. (2012). Mindfulness-based stress reduction training reduces loneliness and pro-inflammatory gene expression in older adults: a small randomized controlled trial. Brain Behav. Immun. 26, 1095–1101 10.1016/j.bbi.2012.07.006 22820409 PMC3635809

[B15] Cullen M. (2011). Mindfulness-based interventions: an emerging phenomenon. Mindfulness 2, 186–193 10.1007/s12671-011-0058-1

[B16] Demarzo M. M. P. Stein P. K. (2012). Mental stress and exercise training response: stress-sleep connection may be involved. Front. Physiol. 3:178 10.3389/fphys.2012.00178 22685438 PMC3368546

[B17] Dimidjian S. Kleiber B. (2013). Being mindful about the use of mindfulness in clinical contexts. Cogn. Behav. Pract. 20, 57–59 10.1016/j.cbpra.2012.02.00616581688

[B18] Duncan L. G. Bardacke N. (2010). Mindfulness-based childbirth and parenting education: promoting family mindfulness during the perinatal period. J. Child Fam. Stud. 19, 190–202 10.1007/s10826-009-9313-7 20339571 PMC2837157

[B19] Edelman D. Oddone E. Z. Liebowitz R. S. Yancy W. S. Jr. Olsen M. K. Jeffreys A. S. (2006). A multidimensional integrative medicine intervention to improve cardiovascular risk. J. Gen. Intern. Med. 21, 728–734 10.1111/j.1525-1497.2006.00495.x 16808774 PMC1924710

[B20] Farb N. A. S. Segal Z. V. Anderson A. K. (2013). Mindfulness meditation training alters cortical representations of interoceptive attention. Soc. Cogn. Affect. Neurosci. 8, 15–26 10.1093/scan/nss066 22689216 PMC3541492

[B21] Fortney L. Taylor M. (2010). Meditation in medical practice: a review of the evidence and practice. Primary Care 37, 81–90 10.1016/j.pop.2009.09.004 20188999

[B22] Garcia M. C. Pompéia S. Hachul H. Kozasa E. H. de Souza A. A. Tufik S. (2014). Is mindfulness associated with insomnia after menopause? Menopause 21, 301–305 10.1097/GME.0b013e31829996fc 23820599

[B23] Garcia-Campayo J. Navarro-Gil M. Andrés E. Montero-Marin J. López-Artal L. Demarzo M. M. P. (2014). Validation of the Spanish versions of the long (26 items) and short (12 items) forms of the Self-Compassion Scale (SCS). Health Qual. Life Outcomes 12:4 10.1186/1477-7525-12-4 24410742 PMC3896764

[B24] Gardner F. L. Moore Z. E. (2012). Mindfulness and acceptance models in sport psychology: A decade of basic and applied scientific advancements. Can. Psychol. 53, 309–318 10.1037/a0030220

[B25] Garland E. L. Gaylord S. A. Boettiger C. A. Howard M. O. (2010). Mindfulness training modifies cognitive, affective, and physiological mechanisms implicated in alcohol dependence: results of a randomized controlled pilot trial. J. Psychoactive Drugs 42, 177–192 10.1080/02791072.2010.10400690 20648913 PMC2921532

[B26] Garland S. N. Campbell T. Samuels C. Carlson L. E. (2013). Dispositional mindfulness, insomnia, sleep quality and dysfunctional sleep beliefs in post-treatment cancer patients. Pers. Indiv. Differ. 55, 306–311 10.1016/j.paid.2013.03.003

[B27] Goldin P. Ziv M. Jazaieri H. Gross J. J. (2012). Randomized controlled trial of mindfulness-based stress reduction versus aerobic exercise: effects on the self-referential brain network in social anxiety disorder. Front. Hum. Neurosci. 6:295 10.3389/fnhum.2012.00295 23133411 PMC3488800

[B28] Grossman P. (2011). Defining mindfulness by how poorly I think I pay attention during everyday awareness and other intractable problems for psychology's (re)invention of mindfulness: comment on Brown et al. (2011). Psychol. Assess. 23, 1034–1040 10.1037/a0022713 22122674

[B28a] Gryffin P. A. Chen W. C. (2013). Implications of t'ai chi for smoking cessation. J. Altern. Complement. Med. 19, 141–145 10.1089/acm.2011.0094 22775366

[B28b] Hagins M. States R. Selfe T. Innes K. (2013). Effectiveness of yoga for hypertension: systematic review and meta-analysis. Evid. Based Complement. Alternat. Med. 2013:649836 10.1155/2013/649836 23781266 PMC3679769

[B29] Hayes-Skelton S. Graham J. (2013). Decentering as a common link among mindfulness, cognitive reappraisal, and social anxiety. Behav. Cogn. Psychother. 41, 317–328 10.1017/S1352465812000902 23218023 PMC3756689

[B30] Hölzel B. K. Lazar S. W. Gard T. Schuman-Olivier Z. Vago D. R. Ott U. (2011). How does mindfulness meditation work? Proposing mechanisms of action from a conceptual and neural perspective. Perspect. Psychol. Sci. 6, 537–559 10.1177/1745691611419671 26168376

[B31] Huang C.-J. Webb H. E. Zourdos M. C. Acevedo E. O. (2013). Cardiovascular reactivity, stress, and physical activity. Front. Physiol. 4:314 10.3389/fphys.2013.00314 24223557 PMC3819592

[B32] Kabat-Zinn J. (2003). Mindfulness-based stress reduction (MBSR). Constr. Hum. Sci. 8, 73–107 Available online at: http://psycnet.apa.org/psycinfo/2004-19791-008

[B33] Kabat-Zinn J. (2005). Full Catastrophe Living: Using the Wisdom of Your Body and Mind to Face Stress, Pain, and Illness. 15th Anniversary Edn New York, NY: Delta Trade Paperback/Bantam Dell

[B34] Khoury B. Lecomte T. Fortin G. Masse M. Therien P. Bouchard V. (2013). Mindfulness-based therapy: a comprehensive meta-analysis. Clin. Psychol. Rev. 33, 763–771 10.1016/j.cpr.2013.05.005 23796855

[B35] Koolhaas J. M. Bartolomucci A. Buwalda B. de Boer S. F. Flügge G. Korte S. M. (2011). Stress revisited: a critical evaluation of the stress concept. Neurosci. Biobehav. Rev. 35, 1291–1301 10.1016/j.neubiorev.2011.02.003 21316391

[B36] Krygier J. R. Heathers J. A. J. Shahrestani S. Abbott M. Gross J. J. Kemp A. H. (2013). Mindfulness meditation, well-being, and heart rate variability: a preliminary investigation into the impact of intensive Vipassana meditation. Int. J. Psychophysiol. 89, 305–313 10.1016/j.ijpsycho.2013.06.017 23797150

[B37] Kuyken W. Byford S. Taylor R. S. Watkins E. Holden E. White K. (2008). Mindfulness-based cognitive therapy to prevent relapse in recurrent depression. J. Consult. Clin. Psychol. 76, 966–978 10.1037/a0013786 19045965

[B38] Libby D. J. Worhunsky P. D. Pilver C. E. Brewer J. A. (2012). Meditation-induced changes in high-frequency heart rate variability predict smoking outcomes. Front. Hum. Neurosci. 6:54 10.3389/fnhum.2012.00054 22457646 PMC3307046

[B39] Malarkey W. B. Jarjoura D. Klatt M. (2013). Workplace based mindfulness practice and inflammation: a randomized trial. Brain Behav. Immun. 27, 145–154 10.1016/j.bbi.2012.10.009 23078984 PMC3528077

[B41] Martarelli D. Cocchioni M. Scuri S. Pompei P. (2011). Diaphragmatic breathing reduces exercise-induced oxidative stress. Evid. Based Complement. Alternat. Med. 2011:932430 10.1093/ecam/nep169 19875429 PMC3139518

[B42] Mitchell J. C. Bach P. A. Cassisi J. E. (2013). The use of structured imagery and dispositional measurement to assess situational use of mindfulness skills. PLoS ONE 8:e70253 10.1371/journal.pone.0070253 23936175 PMC3728095

[B44] Murphy M. J. Mermelstein L. C. Edwards K. M. Gidycz C. A. (2012). The benefits of dispositional mindfulness in physical health: a longitudinal study of female college students. J. Am. Coll. Health 60, 341–348 10.1080/07448481.2011.629260 22686356

[B45] Netz Y. Lidor R. (2003). Mood alterations in mindful versus aerobic exercise modes. J. Psychol. 137, 405–419 10.1080/00223980309600624 14629072

[B46] Nyklíček I. Mommersteeg P. M. C. Van Beugen S. Ramakers C. Van Boxtel G. J. (2013a). Mindfulness-based stress reduction and physiological activity during acute stress: a randomized controlled trial. Health Psychol. 32, 1110–1113 10.1037/a0032200 23527521

[B47] Nyklíček I. van Beugen S. Denollet J. (2013b). Effects of mindfulness-based stress reduction on distressed (Type D) personality traits: a randomized controlled trial. J. Behav. Med. 36, 361–370 10.1007/s10865-012-9431-3 22585012 PMC3710571

[B47a] Ospina M. B. Bond K. Karkhaneh M. Tjosvold L. Vandermeer B. Liang Y. (2007). Meditation practices for health: state of the research. Evid. Rep. Technol. Assess. 1–263 Available online at: http://www.embase.com/search/results?subaction=viewrecord&from=export&id=L350342742 PMC4780968 17764203

[B48] Park T. Reilly-Spong M. Gross C. R. (2013). Mindfulness: a systematic review of instruments to measure an emergent patient-reported outcome (PRO). Qual. Life Res. 22, 2639–2659 10.1007/s11136-013-0395-8 23539467 PMC3745812

[B49] Peressutti C. Martín-gonzález J. M. García-manso J. M. (2011). Does mindfulness meditation shift the cardiac autonomic nervous system to a highly orderly operational state? Int. J. Cardiol. 154, 210–212 10.1016/j.ijcard.2011.10.054 22075417

[B49a] Posadzki P. Jacques S. (2009). Tai chi and meditation: a conceptual (re)synthesis? J. Holist. Nurs. 27, 103–114 10.1177/0898010108330807 Available online at: http://www.embase.com/search/results?subaction=viewrecord&from=export&id=L355102988 19443697

[B50] Prakhinkit S. Suppapitiporn S. Tanaka H. Suksom D. (2013). Effects of buddhism walking meditation on depression, functional fitness, and endothelium-dependent vasodilation in depressed elderly. J. Altern. Complement. Med. [Epub ahead of print]. 10.1089/acm.2013.0205 24372522

[B51] Rosenkranz M. A. Davidson R. J. Maccoon D. G. Sheridan J. F. Kalin N. H. Lutz A. (2013). A comparison of mindfulness-based stress reduction and an active control in modulation of neurogenic inflammation. Brain Behav. Immun. 27, 174–184 10.1016/j.bbi.2012.10.013 23092711 PMC3518553

[B52] Salmoirago-Blotcher E. Crawford S. Carmody J. Rosenthal L. Ockene I. (2011). Characteristics of dispositional mindfulness in patients with severe cardiac disease. J. Evid. Based Complement. Altern. Med. 16, 218–225 10.1177/2156587211405525 22116792 PMC3221306

[B53] Shapiro S. L. Brown K. W. Thoresen C. Plante T. G. (2011). The moderation of Mindfulness-based stress reduction effects by trait mindfulness: results from a randomized controlled trial. J. Clin. Psychol. 67, 267–277 10.1002/jclp.20761 21254055

[B54] Soler J. Cebolla A. Feliu-Soler A. Demarzo M. M. P. Pascual J. C. Baños R. (2014). Relationship between meditative practice and self-reported mindfulness: the MINDSENS composite index. PLoS ONE 9:e86622 10.1371/journal.pone.0086622 24466175 PMC3899282

[B55] Sothmann M. S. Buckworth J. Claytor R. P. Cox R. H. White-Welkley J. E. Dishman R. K. (1996). Exercise training and the cross-stressor adaptation hypothesis. Exerc. Sport Sci. Rev. 24, 267–287 10.1249/00003677-199600240-00011 8744253

[B56] Starkweather A. R. (2007). The effects of exercise on perceived stress and IL-6 levels among older adults. Biol. Res. Nurs. 8, 186–194 10.1177/1099800406295990 17172317

[B57] Stoner L. Lucero A. A. Palmer B. R. Jones L. M. Young J. M. Faulkner J. (2013). Inflammatory biomarkers for predicting cardiovascular disease. Clin. Biochem. 46, 1353–1371 10.1016/j.clinbiochem.2013.05.070 23756129

[B58] Stults-Kolehmainen M. A. (2013). The interplay between stress and physical activity in the prevention and treatment of cardiovascular disease. Front. Physiol. 4:346 10.3389/fphys.2013.00346 24348424 PMC3841719

[B59] Tanay G. Bernstein A. (2013). State Mindfulness Scale (SMS): development and initial validation. Psychol. Assess. 25, 1286–1299 10.1037/a0034044 24059475

[B60] Thayer J. F. Yamamoto S. S. Brosschot J. F. (2010). The relationship of autonomic imbalance, heart rate variability and cardiovascular disease risk factors. Int. J. Cardiol. 141, 122–131 10.1016/j.ijcard.2009.09.543 19910061

[B61] Tsang H. W. H. Chan E. P. Cheung W. M. (2008). Effects of mindful and non-mindful exercises on people with depression: a systematic review. Br. J. Clin. Psychol. 47(Pt 3), 303–322 10.1348/014466508X279260 18237457

[B62] Ulmer C. S. Stetson B. A. Salmon P. G. (2010). Mindfulness and acceptance are associated with exercise maintenance in YMCA exercisers. Behav. Res. Ther. 48, 805–809 10.1016/j.brat.2010.04.009 20488433

[B63] Zgierska A. Obasi C. N. Brown R. Ewers T. Muller D. Gassman M. (2013). Randomized controlled trial of mindfulness meditation and exercise for the prevention of acute respiratory infection: possible mechanisms of action. Evid. Based Complement. Altern. Med. 2013:952716 10.1155/2013/952716 24191174 PMC3804433

